# Degradation Tendency Prediction for Pumped Storage Unit Based on Integrated Degradation Index Construction and Hybrid CNN-LSTM Model

**DOI:** 10.3390/s20154277

**Published:** 2020-07-31

**Authors:** Jianzhong Zhou, Yahui Shan, Jie Liu, Yanhe Xu, Yang Zheng

**Affiliations:** 1 School of Hydropower and Information Engineering, Huazhong University of Science and Technology, Wuhan 430074, China; jz.zhou@hust.edu.cn (J.Z.); jie_liu@hust.edu.cn (J.L.); yh_xu@hust.edu.cn (Y.X.); 2 School of Power and Mechanical Engineering, Wuhan University, Wuhan 430072, China; zhengyang@whu.edu.cn

**Keywords:** pumped storage unit, Gaussian process regression, integrated degradation index, convolutional neural network, long and short memory neural network, degradation tendency prediction

## Abstract

Accurate degradation tendency prediction (DTP) is vital for the secure operation of a pumped storage unit (PSU). However, the existing techniques and methodologies for DTP still face challenges, such as a lack of appropriate degradation indicators, insufficient accuracy, and poor capability to track the data fluctuation. In this paper, a hybrid model is proposed for the degradation tendency prediction of a PSU, which combines the integrated degradation index (IDI) construction and convolutional neural network-long short-term memory (CNN-LSTM). Firstly, the health model of a PSU is constructed with Gaussian process regression (GPR) and the condition parameters of active power, working head, and guide vane opening. Subsequently, for comprehensively quantifying the degradation level of PSU, an IDI is developed using entropy weight (EW) theory. Finally, combining the local feature extraction of the CNN with the time series representation of LSTM, the CNN-LSTM model is constructed to realize DTP. To validate the effectiveness of the proposed model, the monitoring data collected from a PSU in China is taken as case studies. The root mean square error (RMSE), mean absolute error (MAE) and mean absolute percentage error (MAPE) obtained by the proposed model are 1.1588, 0.8994, 0.0918, and 0.9713, which can meet the engineering application requirements. The experimental results show that the proposed model outperforms other comparison models.

## 1. Introduction

A pumped storage unit (PSU) operates under the combination of different conditions, which may cause equipment wear, degradation, and fault issues [1]. Degradation tendency prediction (DTP) is essentially a time-series prediction problem, namely predicting the future degradation propagation using the history and current monitoring data [2]. Accurate DTP can not only discover abnormal operating conditions, but also improve the reliability and stability of a PSU [3]. Thus, it is crucial to research DTP under different conditions to reduce failure rate, maintenance cost, and downtime [4,5,6].

In general, DTP mainly includes physics-based and data-based methods [7]. Physics-based methods would face more restrictions due to the heavy dependence on the physical rules and domain expertise. Nevertheless, without much expertise, data-driven methods can conduct DTP by mining the historical monitoring data and have attracted much attention in machinery prognostics [8]. They can be applied to the prognosis of complicated systems whose degradation process are difficult to analyze through physics methods [9]. Therefore, it is reasonable to conduct the data-driven DTP of PSU. Summarizing relevant literature, data-driven prognosis methods can mainly be divided into three stages, i.e., health state model establishment, performance degradation index (PDI) construction, and DTP modeling [10].

The determination of monitoring data that can best reflect the status of equipment is the key to establishing the health state model. The monitoring data, such as vibration, working head (*H*), active power (*P*), guide vane opening (*G*) etc. can be adopted to characterize the health state [3,11]. They comprise important information about the development of degradation tendency. For example, Gebraeel et al. utilized the collected vibration signal to analyze the state of a rotating bearing [12]. Fu et al. established the trend prediction model with the water guide vibration of a hydropower unit [13]. Hu et al. used working head and active power to predict the degradation tendency of hydropower unit [14]. However, the one-dimensional monitoring data may ignore some essential information. Moreover, only considering the working head and active power is insufficient to characterize the degradation level in reference [14]. Therefore, multi-dimensional monitoring data (*H*, *P*, *G*) should be considered to establish an effective health state model.

With the elapse of time, a large amount of monitoring data from different objects can be collected for analysis. Thus, it is essential to construct an appropriate PDI to make full use of these data, which is considered as the preliminary step of the subsequent degradation prediction [15]. To quantitatively reflect the degradation level, researchers have been focusing on constructing a PDI. For instance, Guo et al. constructed one-dimensional PDI utilizing the recurrent neural network (RNN) to predict remaining useful life of bearing [16]. An et al. adopted a PDI with the upper bracket horizontal vibration data to analyze the degradation trend of hydropower unit [17]. However, these PDIs are constructed mainly with single sensor data or a single object, which cannot comprehensively assess the degradation of the entire equipment. Therefore, the construction of a comprehensive degradation index utilizing multi-dimensional monitoring data from multiple objects, in simple form, is a challenge in the process of DTP. 

DTP aims to obtain the future degradation level and provide a sufficient data base for decision-making. The research on tendency prediction has been widely developed, and the related research on hydropower unit is also gradually increasing. For instance, Fu et al. used the least squares support vector regression (LS-SVR) model to predict the vibration tendency of hydropower unit [18]. An et al. solved the DTP of hydropower unit with radial basis function neural network (RBFNN) and grey theory [17]. In the above methods, the parameters of LS-SVR affect its performance in dealing with constrained optimization problem. The structure and parameter of RBFNN are generally specified manually or initialized randomly, which weakens its generalization ability. Due to the synthesis of useful expressions and nonlinear transformations, deep learning has been drawn much attention and successfully made great progress in the field of prognostic and health management [19,20,21,22]. For example, Lu et al. utilized the convolutional neural network (CNN) for in situ fault diagnosis [23]. Xiang and Qin applied long short term memory (LSTM) network for gear remaining life prediction [24,25]. The CNN can extract interrelations among input data utilizing convolutional filters, while LSTM can process time series information by characterizing long-term dependency. Over recent years, the combined CNN and LSTM was developed to extract spatial and temporal features. For instance, Kim proposed a CNN-LSTM method to predict the residential energy consumption [26]. An et al. predict remaining useful life for milling tool by designing the convolutional and stacked LSTM network [27]. However, the deep learning techniques, especially the combination of CNN and LSTM, have rarely been applied for DTP for PSU. Moreover, owing to the nonlinearity and strong fluctuation of degradation tendency, a simple combination network is usually ineffective.

Based on the above discussion, this paper is motivated by the following aspects: (1) the effects of condition parameters should be comprehensively considered in health state model; (2) the integrated degradation index (IDI) should be constructed to quantify the degradation level; (3) the combination models like CNN and LSTM can achieve high accuracy in prediction. Thus, a hybrid model is proposed for DTP of PSU in this paper. More specifically, the health state model of PSU is constructed with the monitoring data (*H*, *P*, *G*) and Gaussian process regression (GPR) at first. It can fully reveal the relationship between the PSU’s operating status and the monitoring data. In order to accurately quantify the degradation level of PSU, the IDI is built with entropy weight (EW) and PDIs from multiple objects, such as the lower bracket and the lower guide of PSU. Furthermore, to enhance the performance of the prediction model, the CNN-LSTM model is developed for DTP. The main contributions are highlighted as follows:

(1) Considering the multi-dimensional monitoring data such as working head (*H*), active power (*P*), and guide vane opening (*G*), the health state model is established.

(2) In order to characterize a PSU’s performance, the IDI is defined using EW theory and PDIs from multiple objects.

(3) For capturing the nonlinear characteristic from IDI series, a hybrid CNN-LSTM model is applied for DTP, where CNN can extract local features, LSTM can map the features into separable spaces to produce prediction results.

The rest of this paper is organized below. Section 2 reviews the related knowledge about GPR, EW, CNN and LSTM. The framework of the proposed DTP model is presented in Section 3. Then, Section 4 illustrates the engineering application and analysis with the proposed model. Finally, the conclusions are given in Section 5.

## 2. Background Knowledge

### 2.1. Gaussian Process Regression

With the flexibility in describing modeling uncertainty, GPR outperforms in fitting and regression problems [28]. GPR can model time series with Gaussian prior, which is determined by the kernel function $K(x_{i},x_{j})$ and mean function [29,30]. The process of GPR is described as follows:

(1) $$y=f\left(x_{n}\right)+\xi_{n},$$

where *x* is the input and *y* is the output. $f\left(x_{n}\right)$ is a hidden function. $\xi_{n}$ is the noise.

Given the data $D=\left[\left(x^{*},y^{*}\right)\right]$, the joint distribution of the training set *y* and y* can be obtained according to the Bayesian principle:

(2) $$\left[\begin{array}{l} y\\ y^{*} \end{array}\right]=N\left(0,\left[\begin{array}{cc} K(X,X)+\sigma_{n}^{2}I & K\left(X,x^{*}\right)\\ K\left(x^{*},X\right) & k\left(x^{*},x^{*}\right) \end{array}\right]\right).$$

After the training set and testing set are given, the posterior probability distribution of y* is expressed as:

(3) $$y^{*}|X,y,X^{*}\sim N\left[{\bar{y}}^{*},\mathrm{cov}\left(y^{*}\right)\right],$$

(4) $${\bar{y}}^{*}=K\left(x^{*},X\right){\left(K(X,X)+\sigma_{n}^{2}I\right)}^{-1}y,$$

(5) $$Cov(y^{*})=k\left(x^{*},x^{*}\right)-K\left(x^{*},X\right){\left[K(X,X)+\sigma_{n}^{2}I\right]}^{-1}K\left(X,x^{*}\right),$$

where ${\bar{y}}^{*}$ and $Cov(y^{*})$ are the fitting value and variance, respectively.

### 2.2. Convolutional Neural Network

Through layer-by-layer convolution operation, CNN can extract the spatial features hidden inside the data [31]. The features can be further applied for classification or regression. Different topologies have a direct impact on the network’s fitting ability and generalization performance. Generally, CNN has achieved great success in processing 2D data, which has three dimensions: width, height, and number of channels. Particularly, the 1D CNN can be regarded as a special case, when the height of the input data is regarded as 1 [32]. Thus, 1D CNN is applied for capturing the spatial features of degradation tendency.

The convolution layer can be considered as a filter of multiple input data to extract features. The input is processed by convolutional operation before passing to the next layer [33].

(6) $$y_{k}=\sigma *\left(W*x_{k}+b\right),$$

where *W* is the filter weight, *b* denotes a bias parameter, and σ represents the underlying activation function.

### 2.3. Long Short-Term Memory Neural Network

With recurrent connections in hidden layers, RNN can consider contextual information in history input, which makes it suitable for the analysis of sequential data [34]. However, due to the gradient explosion and vanishing, RNN is unable to represent long-term dependency characteristics of sequence data [35]. In contrast, as one of the most popular variants of RNN, the LSTM neural network can avoid such drawbacks by using hidden memory. It extends RNN with three types gates: the input gate determining whether the current input should be stored, the forget gate controlling whether the historical information is forgotten of the cell memory, and the output gate determining the information that flows into node output [36].

With the special memory structure and gated designing, LSTM has a better ability to learn long-term dependency. The structure of LSTM can be described by the following equations [36,37]:

(7) $$\left\{ \begin{array}{l} i_{t}=\sigma \left(W_{i}\cdot \left[h_{t-1},x_{t}\right]+b_{i}\right)\\ {\tilde{c}}_{t}=\tanh\left(W_{c}\cdot \left[h_{t-1},x_{t}\right]+b_{c}\right)\\ c_{t}=f_{t}*c_{t-1}+i_{t}*{\tilde{c}}_{t} \end{array}\right.,$$

(8) $$f_{t}=\sigma \left(W_{f}\cdot \left[h_{t-1},x_{t}\right]+b_{f}\right),$$

(9) $$\left\{ \begin{array}{l} o_{t}=\sigma \left(W_{o}\cdot \left[h_{t-1},x_{t}\right]+b_{o}\right)\\ h_{t}=o_{t}*\tanh\left(c_{t}\right) \end{array}\right.,$$

where *i_t_*, *f_t_*, *c_t_*, and *o_t_* represent the input gate, forget gate, cell activation vectors, and output gate, respectively.

## 3. Degradation Tendency Prediction Model of PSU

For modeling the highly nonlinear dynamics and estimating degradation tendency, a hybrid DTP model of PSU based on IDI construction and CNN-LSTM is proposed, which considers multi-dimension monitoring data from multiple objects. The proposed model systematically has a fine mix of mathematical statistics and intelligent prediction technology. This section consists of four parts: (1) analyze the monitoring data and construct the health model; (2) generate IDI series of degradation tendency using EW theory and PDIs; (3) construct the CNN-LSTM predict model; and (4) illustrate the general procedure of the proposed model.

### 3.1. Analyze and Establish the Health State Model

With the accumulation of running time, PSU gradually experiences the process of health, degradation and failure. The gradual nature makes the degradation tendency predictable. Through in-depth analysis of historical data, it can be found that the most representative monitoring data are working head (*H*), active power (*P*) and guide vane opening (*G*) of PSU. By constructing the health state model with GPR, the mapping between monitoring data (*H*, *P*, *G*) and operation status data (*F*) can be obtained.

#### 3.1.1. Analyze the Monitoring Data

With the continuous expansion of online monitoring systems, the relationship between monitoring data and operating state can be effectively mined to determine the health state of PSU. Through data visualization, the relationship between monitoring data (*H*, *P*, *G*) and operation status data (*F*) is analyzed. The sensitive monitoring data and measured operation status data are selected as input and output of the health state model, respectively.

#### 3.1.2. Establish Health State Model

Based on the above comprehensive analysis, it can be found that the monitoring data (*H*, *P*, *G*) are the three main factors affecting the operation of PSU. Therefore, a standard multi-dimensional health state model is constructed with GPR. The model can accurately describe the health state with the monitoring data. The recorded monitoring data (*H*, *P*, *G*) are brought into GPR and the corresponding operation status data (*F*) is the output. The mapping relationship can be expressed as follows:

(10) F(t)=f(H(t), P(t), G(t)).

In health state model establishment stage, the correlation (*R*) is utilized as the criterion of model fitting [37].

(11) $$R=\frac{\frac{1}{N}\overset{N}{\underset{i=1}{\sum }}\left(r(t)-\bar{r}(t))(c(t)-\bar{c}(t)\right)}{\sqrt{\frac{1}{N}\overset{N}{\underset{i=1}{\sum }}{\left(r(t)-\bar{r}(t)\right)}^{2}}\times \sqrt{\frac{1}{N}\overset{N}{\underset{i=1}{\sum }}{\left(c(t)-\bar{c}(t)\right)}^{2}}},$$

where c(t) is the model fitting value, r(t) is the actual value of PSU, *N* is the length of the data, *t* is the operating time, while $\bar{r}(t)$ and $\bar{c}(t)$ represent the average of the actual value and the fitting value, respectively.

### 3.2. Construct IDI for PSU

In order to effectively quantify the degradation level of PSU, a comprehensive PDI should be constructed. For each object with available data, its PDI can be constructed based on the health state model. However, the PDI of a single object cannot represent the degradation degree of the whole PSU. With the help of available PDI of multiple objects and EW theory, an IDI is developed to comprehensively describe the health state of PSU.

#### 3.2.1. Construction of PDI

The monitoring data (*H*, *P*, *G*) are taken as input of the health state model. The standard value of health state *V*(*t*) can be obtained. Then, comparing *V*(*t*) with the measured operation status data *F*(*t*) of a PSU, the PDI of the object is developed as follows:

(12) $$PDI(t)=\frac{\left|F(t)-V(t)\right|}{V(t)}\times 100\%.$$

#### 3.2.2. Construct IDI with PDIs and EW Theory

Since the PDIs from each objects have different effects on the health state of PSU, its information entropy is also different [38]. The weight identified by entropy can indicate the amount of useful information contained in PDIs [39]. Therefore, the IDI using EW theory and PDIs of different objects is defined with all effective information retained, characterizing the health state of PSU. The specific process is described as follows:

With the obtained PDIs of different objects, the input matrix of entropy is given in Equation (13):

(13) $$X_{m\times n}=\left[\begin{array}{l} X_{11}\;X_{12}\;\ldots \;X_{1j}\\ X_{21}\;X_{22}\;\ldots \;X_{2j}\\ \;\;\;\vdots \;\;\;\;\;\vdots \;\;\;\;\;\;\;\;\vdots \\ X_{i1}\;\;X_{i2}\;\ldots \;X_{ij} \end{array}\right],$$

where *X_ij_* represents the index in the *i*-th data sequence of the *j*-th PDI, *i* = 1, 2, 3 …, *m*, *j* = 1, 2, 3 …, *n*.

Since the obtained PDIs have the same dimension, the process of normalizing is as follows:

(14) $$Y_{ij}=\frac{X_{ij}-X_{\min}}{X_{\max}-X_{\min}},$$

where *Y_ij_* is the normalized value corresponding to *X_ij_*.

Then, the entropy of normalized PDIs can be calculated by [40]:

(15) $$p_{i}=Y_{ij}/\overset{n}{\underset{i=1}{\sum }}Y_{ij},$$

(16) $$E_{i}=-\ln{\left(n\right)}^{-1}\overset{n}{\underset{i=1}{\sum }}p_{i}\ln p_{i},$$

where $p_{i}$ is the probability of the PDIs series and $E_{i}$ is value of the information entropy.

According to the entropy results, the entropy weights $W_{1}$, $W_{2}$, … $W_{k}$ of each PDI can be calculated [41]:

(17) $$W_{i}=\frac{1-E_{i}}{k-\sum E_{i}}(i=1,2,\cdots k),$$

(18) $$\overset{n}{\underset{j}{\sum }}W_{j}=1,\;\;\;W=\left(W_{1},W_{2},W_{3},\ldots ,W_{j}\right),$$

where $W_{i}$ represents the weight corresponding to the PDI, *W* is the weight matrix, and *k* is the number of PDIs series.

Finally, the IDI can be obtained as follows:

(19) $$IDI=W\cdot X_{m\times n}.$$

Note that the value of IDI varies from 0 to 1, “0” for health, and “1” for failure. In essence, the process of IDI can be regarded as the multi-dimensional data fusion, which can effectively and comprehensively describe the health state of PSU.

### 3.3. Predict the Degradation Tendency of PSU with CNN-LSTM

To improve prediction performance, a hybrid CNN-LSTM model is proposed for DTP of PSU. It takes the constructed IDI series and the future data of degradation tendency as the input and output, respectively.

#### 3.3.1. The Structure of CNN-LSTM

**Figure 1:**
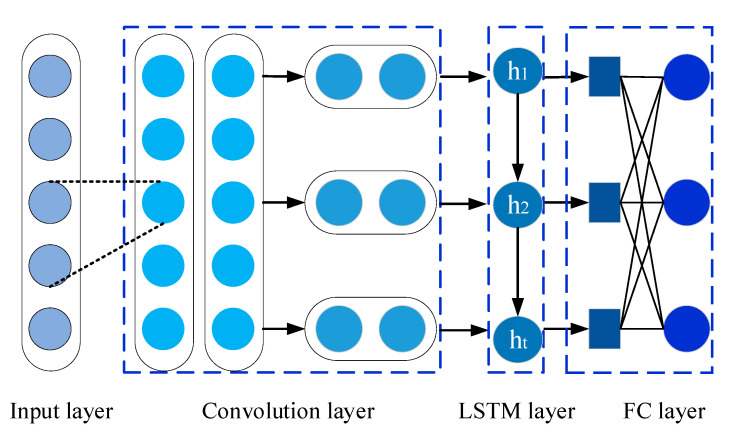
The structure of the proposed CNN-LSTM network.

In CNN-LSTM network, the CNN is applied to extract advanced spatial features from IDI, and the LSTM can investigate the relationships between historical inputs and current health state. The structural schematic of CNN-LSTM is illustrated in Figure 1. Specifically, the input layer is constructed from IDI time series variables. Next, two one-dimensional convolutional layers are built for extracting spatial features. After that, a LSTM layer is added to extract temporal nonlinearity inside IDI. Finally, two fully connected layers are applied to produce the final prediction results of the IDI.

In order to ensure that the proposed network has better accuracy and generalizability, an Adam optimizer is applied for minimizing the total loss [42]. The biases and weights are updated based on the gradient of the loss function. Meanwhile, the maximum training epochs is 200 and the initial learning rate is 0.01. To avoid possible over-fitting, the dropout rate is 0.2 in fully connected layer (FC). Meanwhile, the loss function is mean square error (MSE) in training as follows:

(20) $$\mathrm{MSE}=\frac{1}{K}\overset{K}{\underset{k=1}{\sum }}{\left(y_{k}-{\hat{y}}_{k}\right)}^{2},$$

where ${\hat{y}}_{k}$ is the output, $y_{k}$ is the real value, *K* is number of the training data.

#### 3.3.2. Evaluation Criterion

To evaluate the effectiveness of prediction model, three common indexes such as root mean square error (RMSE), mean absolute error (MAE) and mean absolute percentage error (MAPE) are employed for evaluation [43].

(21) $$RMSE=\sqrt{\frac{1}{N}\overset{N}{\underset{i=1}{\sum }}{\left(y_{i}-{\hat{y}}_{i}\right)}^{2}},$$

(22) $$MAE=\frac{1}{N}\overset{N}{\underset{i=1}{\sum }}\left|y_{i}-{\hat{y}}_{i}\right|,$$

(23) $$MAPE=100\%\times \frac{1}{N}\overset{N}{\underset{I=1}{\sum }}\left|\frac{y_{i}-{\hat{y}}_{i}}{y_{i}}\right|,$$

where $y_{i}$ and ${\hat{y}}_{i}$ are the actual and predicted values, respectively.

Additionally, the improved percentage of the above indexes is applied for evaluating different models quantitatively. The definition of improved percentage $P_{\mathrm{index}\;}$ is defined as follows:

(24) $$P_{\mathrm{index}\;}=|\frac{v_{A}-v_{B}}{v_{A}}|\times 100\%,$$

where $v_{A}$ and $v_{B}$ are the index values of the comparative model and the proposed model, respectively.

### 3.4. The Proposed Degradation Tendency Prediction Model and Framework

**Figure 2:**
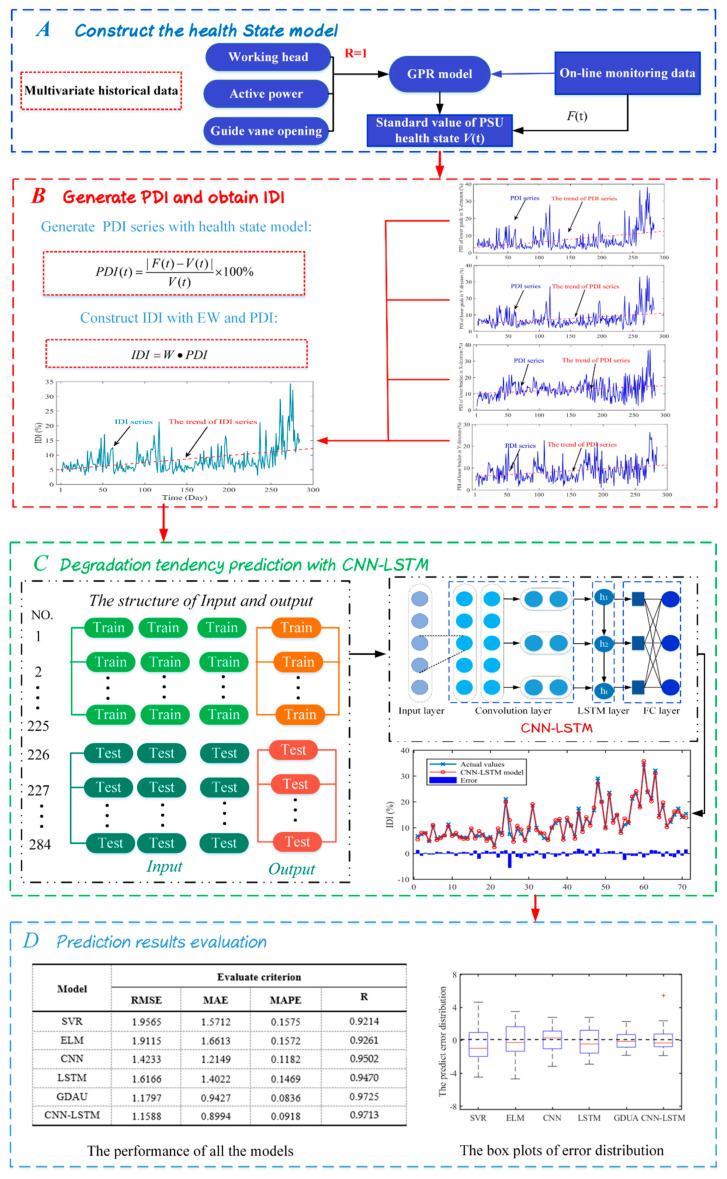
The flowchart of the proposed DTP model.

In this section, a hybrid model is presented that includes the health state model establishment, IDI construction with PDIs and EW theory, CNN-LSTM predicting model and predicting results evaluation. The main steps are displayed in Figure 2. The more specific processes are as described below.

Step A: Construct the health state model

Massive monitoring data of PSU under different conditions are analyzed in detail to determine the health state model of PSU. The sensitive condition parameters such as working head (*H*), active power (*P*), and guide vane opening (*G*) are selected to build health model with GPR. This modeling method can more realistically reflect the effect of condition parameters on the state of PSU.

Step B: Generate PDI and obtain IDI

Real-time monitoring data of PSU can be substituted into the health model. The standard health state value *V*(*t*) is obtained. The standard *V*(*t*) is compared with the real value of the monitoring data *F*(*t*). Then PDI can be defined by Equation (12). With the obtained PDIs of different objects and EW theory, the IDI is defined with all effective important retained, characterizing the health state of PSU.

Step C: Degradation tendency prediction with CNN-LSTM

Due to the nonlinearity of degradation tendency series, simple network prediction is generally not effective. In order to obtain more accurate prediction results of degradation tendency, we constructed the input and output structure and proposed a hybrid CNN-LSTM model. The hybrid model fuses the advantages of CNN in local features extraction and the expression of LSTM on time series.

Step D: Prediction results evaluation

Prediction effectiveness can be measured not only by the square sum of predicting error, but also by the mean squared deviation of the error. In this study, three common evaluation criteria are employed to assess the prediction performance, in particular, RMSE, MAE, and MAPE. In addition, the error distributions and Taylor diagram are also employed to compare the performance of the proposed model more intuitively.

## 4. Engineering Application and Analysis

To validate the proposed DTP model, the experiments were conducted with the dataset collected from PSU, which is located in Jingxing County, Hebei Province of China. The PSU is equipped with a single-stage mixed-flow reversible pump-turbine unit, whose capacity of 250 MW. The maximum turbine/pump head is 346 m, and the minimum turbine/pump head is 291 m. The rated head of turbine is 305 m, and the rated speed of PSU is 333.3 r/min. The experiments applied to the dataset were conducted in MATLAB R2016a software with CPU 2.5 GHz and RAM 32 GB.

The TN8000 condition monitoring system was installed in PSU [44]. It contains local sensors, data acquisition and central web server. It can collect up to 4 key phase signals, 24 vibration/swing signals, 12 static signals and 255 process signals obtained by Modbus communication. The central web server can store and manage the data from data acquisition equipment. The data monitored from TN8000 system are illustrated in Table 1.

The TN8000 system acquires vibration and swing data with Bently 330505 sensors and 3300 sensors, which follows the standard of ISO/IEC7498-IEEE/ANSI 802 [45]. The sensors are a kind of electric eddy current sensor of 3300 series, whose output is 4–20 mA or 1–5 V. In addition, some data like active power and guide vane opening are directly taken from the PSU through hard wiring. And the data like working head needs to be read through the computer supervise and control system. Meanwhile, with the complicated structure and frequent switching of operating conditions, it may easily lead to different monitoring data with different time intervals. Hence, the monitoring data that meet the average time interval were chosen for analysis, which is in line with engineering practice. The corresponding *P*, *H* and *G* are recorded to construct data vectors F (*H*(*t*), *P*(*t*), and *G*(*t*)) from 31 July 2008 to 25 December 2009. They were selected to build the health state model. Then, the PDI from 2 January 2010 to 15 December of 2011 can be obtained.

### 4.1. Data Collection

**Figure 3:**
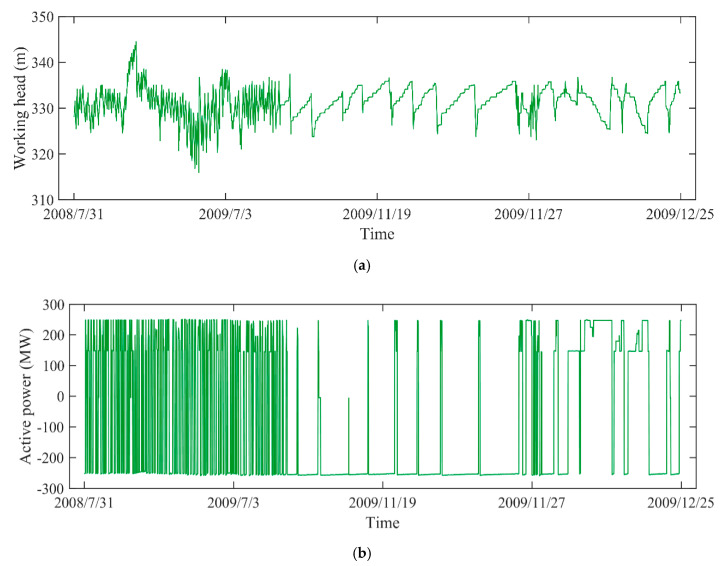

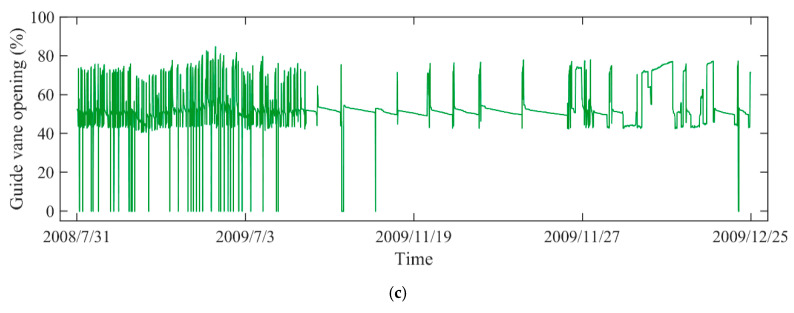
The monitoring data of PSU: (**a**) working head, (**b**) active power, (**c**) guide vane opening.

**Figure 4:**
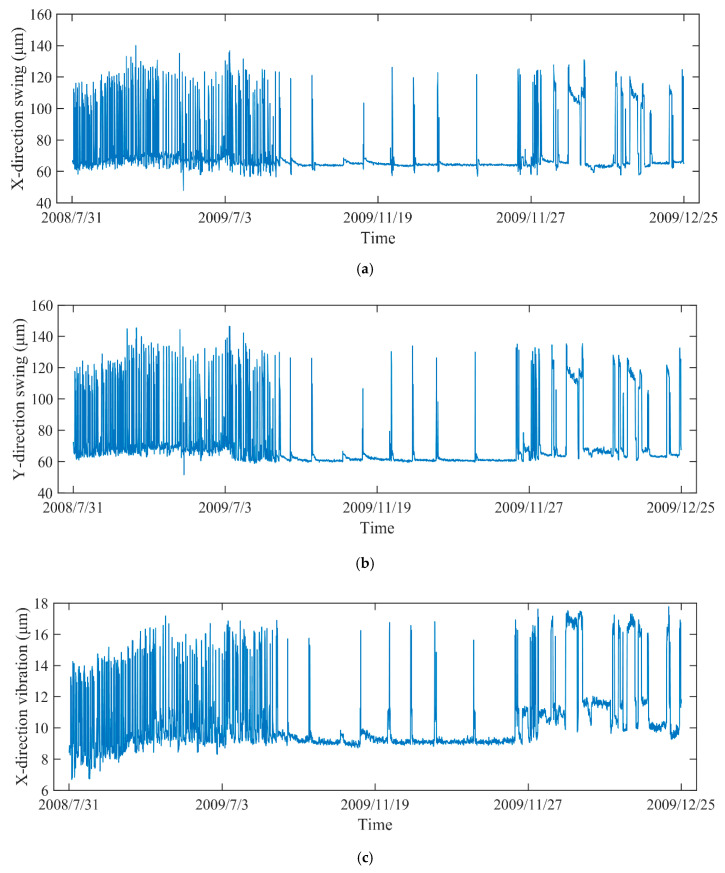

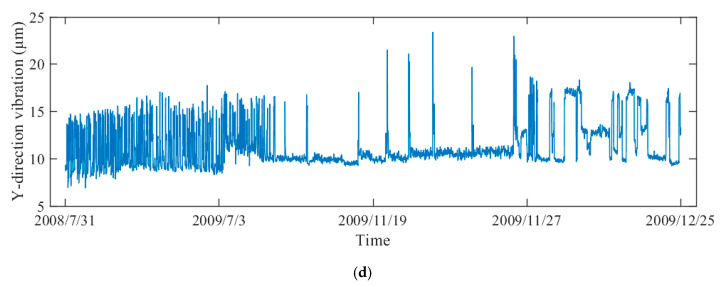
The operation status data of different objects: (**a**) the swing data in X-direction, (**b**) the swing data in Y-direction, (**c**) the vibration data in X-direction, (**d**) the vibration data in Y-direction.

In engineering application, the vibration and swing signals are usually applied for evaluating the status of equipment. To comprehensively quantify the degradation level, the vibration and swing data are selected as for DTP from different objects, such as the vibration in X-direction and Y-direction of lower bracket, the swing data in X-direction and Y-direction of lower guide. The corresponding monitoring data (*H*, *P*, *G*) are recorded as presented in Figure 3. The monitoring data from 31 July 2008 to 25 December 2009 were used to construct the health state model. With the data from 2 January 2010 to 15 December of 2011, the IDI was then obtained based on EW theory and PDIs. As illustrated in Figure 4, due to the integration of various working conditions, including pumping, no-load, and generating operations, the vibration and swing data are strongly non-linear and non-stationary.

### 4.2. Establish and Verify the Health State Model for PSU

#### 4.2.1. Monitoring Data Analysis

**Figure 5:**
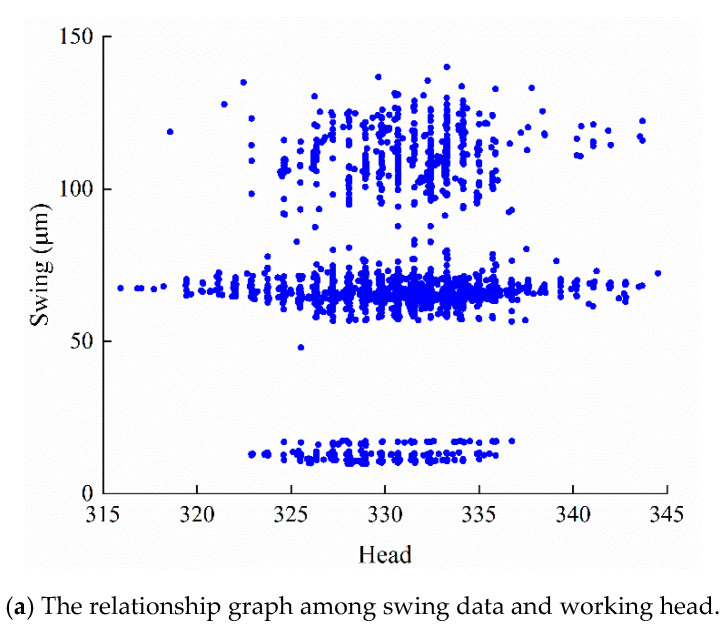

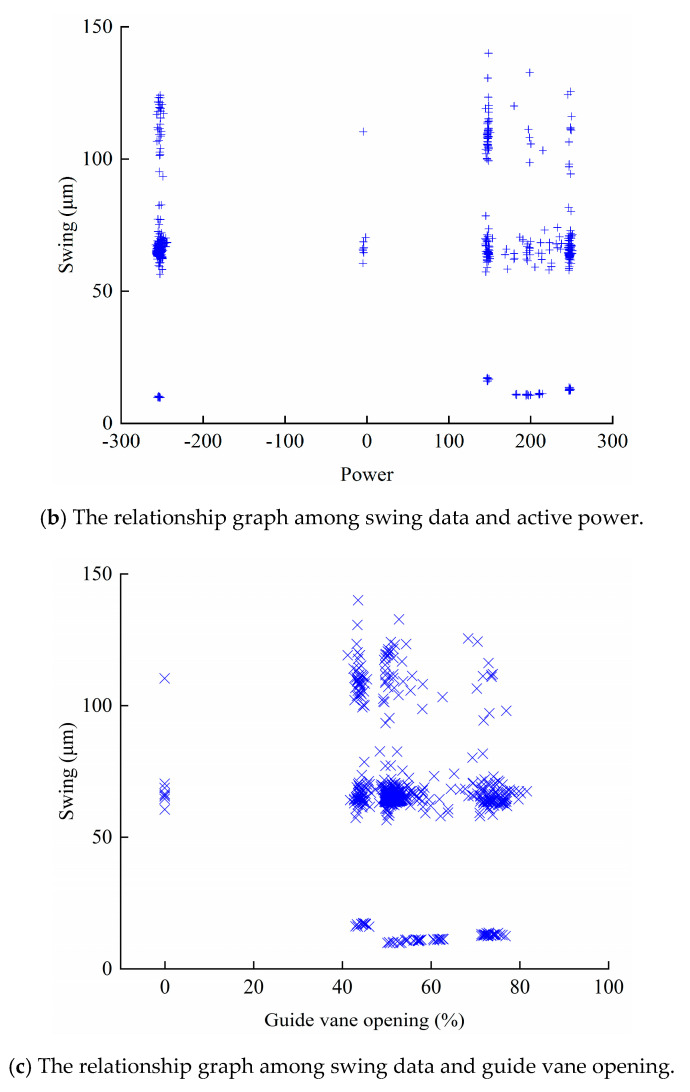
The relationship graph among swing data in Y direction and monitoring data (*H*, *P*, *G*): (**a**) working head, (**b**) active power and (**c**) guide vane opening.

To further analyze the relationship between monitoring data (*H*, *P*, *G*) and operation status data (*F*), the data visualization is utilized. With the reversible operation and frequent condition switching, the monitoring data covers different working conditions of PSU. In addition, the obtained relationships are different among the four kinds of vibration and swing signals. For simplicity, the X-direction swing data of the lower guide were selected for detailed discussion. The relationship diagrams are shown in Figure 5.

Specially, Figure 5a illustrates the relation diagram between *H* and swing data, it can be seen that the distribution of *H* is mainly concentrated between 315 m and 345 m. Figure 5b is the relationship between *P* and swing data. There are three parts of swing data: the generating condition dataset, the no-load condition dataset and pumping condition dataset. The data are mainly distributed in 0 MW and −250 MW under no-load condition and pumping condition, while the distributions in generating condition are mainly concentrated in 250 MW, 200 MW and 150 MW. Figure 5c shows the relationship between *G* and swing data. The data can be divided into two parts, including the guide vane closing dataset and the guide vane opening dataset. The guide vane opening dataset is mainly distributed at 40–90% opening.

#### 4.2.2. Health State Model Establishment

Based on the above analysis, it can be seen that there is a clear mapping relationship between monitoring data (*H*, *P*, *G*) and operation status data (*F*) of PSU. And the vibration and swing data are greatly affected by the monitoring data (*H*, *P*, *G*). Therefore, the health state model was constructed with GPR and the operation status data (*F*). The 4000 data were selected from 31 July 2008 to 25 December 2009. Among them, three out of four samples were used for the health state model training, while the remainder was utilized for testing. The parameters of GPR are shown in Table 2.

**Figure 6:**
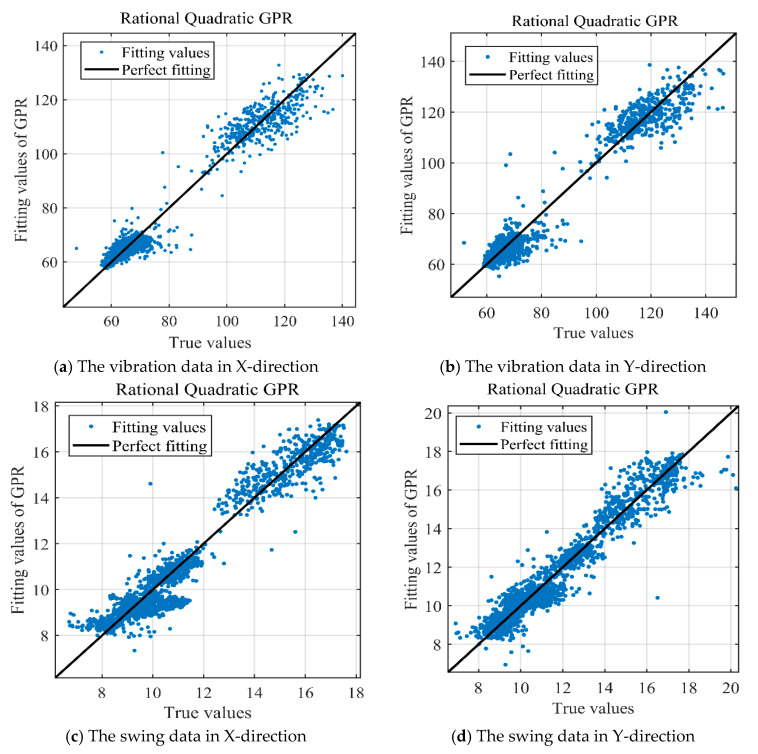
The fitting results of GPR: (**a**) the vibration data in X-direction, (**b**) the vibration data in Y-direction, (**c**) the swing data in X-direction, (**d**) the swing data in Y-direction.

The fitting results of the swing and vibration data are shown in Figure 6. In the model establishment stage, the values of R are 0.9714, 0.9716, 0.9634, and 0.9539, respectively. This means that the health state model can represent the original PSU at a high level of confidence.

### 4.3. Construct IDI with PDI and EW Theory

#### 4.3.1. Construction of PDI

**Figure 7:**
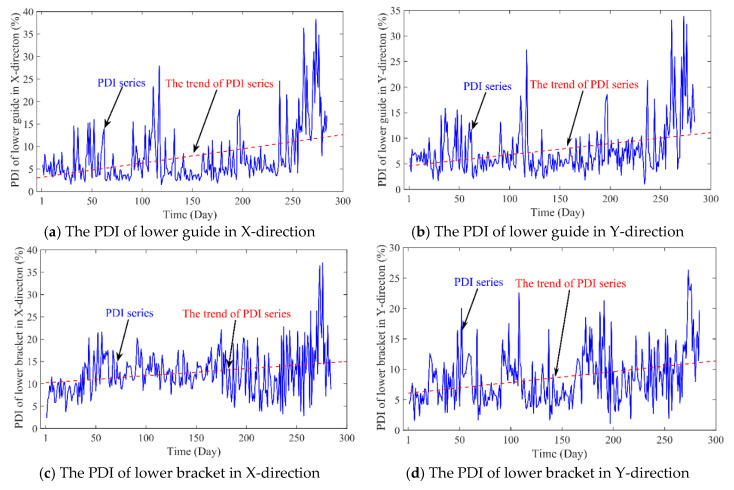
The PDI obtained from different objects: (**a**) the PDI of lower guide in X-direction, (**b**) the PDI of lower guide in Y-direction, (**c**) the PDI of lower bracket in X-direction, (**d**) the PDI of lower bracket in Y-direction.

The measured data from 2 January 2010 to 15 December of 2011 were adopted to construct the PDI of PSU. Given the operation status data *F*(*t*), the monitoring data (*H*, *P*, *G*) in the same state are brought into the established health model. Then, the standard value of operation status *V*(*t*) can be obtained. So, the PDI series can be obtained through Equation (12). As shown in Figure 7, there are four PDI series from vibration data in X-direction and Y-direction of lower bracket, swing data in *X*-direction and Y-direction of lower guide. The daily mean value of PDIs tends to increase, which means the performance of PSU gradually deteriorates overtime. However, it is insufficient to randomly select any of the obtained PDIs to represent the degradation tendency of the entire PSU.

#### 4.3.2. Construct IDI with PDIs and EW Theory

**Figure 8:**
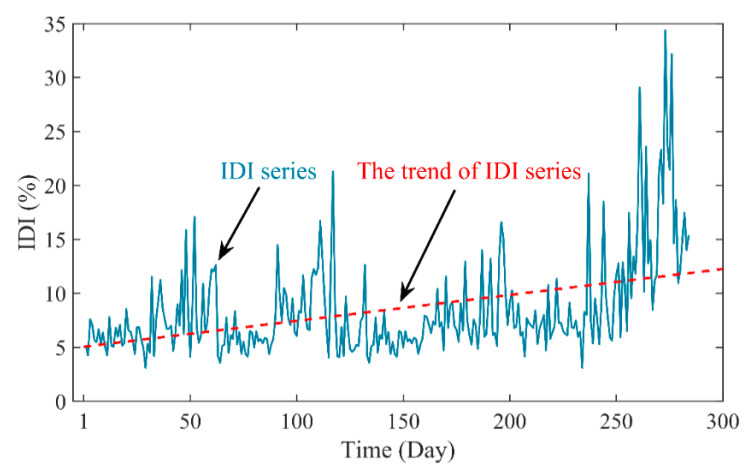
The IDI series of the performance degradation tendency.

To reduce the impact of PDIs irregular fluctuations and obtain the comprehensive PDI series of PSU, an integrated strategy with EW theory is developed as described in Section 3.2. First, the weight is calculated by EW for the four PDI series. The entropy and its weight values of the PDIs can be obtained as shown in Table 3. The values of entropy are within the range of 0.95–1, which indicates that these PDI series can represent the degradation degree of the PSU to a certain extent. Then, as illustrated in Figure 8, IDI series can be obtained with the PDIs and its weight based on Equation (19). The entropy value of IDI series is 0.9502. This means the generated IDI series can retain the complete information in the PDIs and reduce the complexity of PDIs series. In summary, the IDI can be used as an effective indictor to evaluate the degradation level of PSU. 

### 4.4. Degradation Tendency Prediction of PSU with CNN-LSTM

With the IDI series of the degradation tendency, the proposed CNN-LSTM model is applied for DTP. The first 225 points were taken as the training set and the rest were as the testing set. Taken the fixed length data as input vectors, the CNN extracts the non-linear spatial features from the input, and the LSTM can model relationships between historical data and current health state. Finally, the results of DTP are obtained. Table 4 illustrates the structure parameters of CNN-LSTM, which includes the activation, the number of kernel size, stride, and the filter in convolution layer.

**Figure 9:**
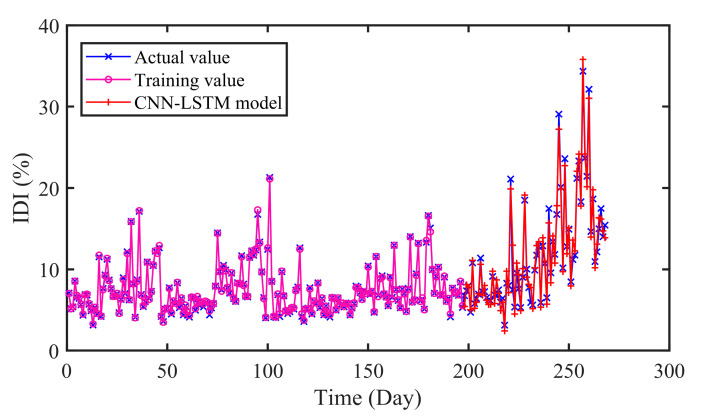
The predicted results of CNN-LSTM model.

With the proposed CNN-LSTM model, the prediction result is obtained as illustrated in Figure 9. The performance of CNN-LSTM model is generally satisfactory. In addition to the local abrupt points, the prediction values and the actual values of the degradation time series fit well. Owing to the strong nonlinear learning and fitting ability, the proposed model can better predict the degradation tendency.

### 4.5. Results and Analysis

To verify the nonlinear characterization ability of CNN-LSTM, two types of comparison experiments are carried out. Specifically, the first type includes support vector regression (SVR)-based model and extreme learning machine (ELM)-based model. The second includes CNN-based model, LSTM-based model and gated dual attention unit neural network (GDAU) [46]. Furthermore, the analysis and discussion are conducted for a more comprehensive evaluation of the proposed model.

#### 4.5.1. Comparison of SVR and ELM Prediction

SVR is an extended application of support vector machine from classification to regression. The parameters of SVR are searched by grid search (GS) method in this section. As a feedforward neural network with single hidden layer, ELM has been widely studied for its fast convergence speed. And the optimal number of hidden layer nodes is determined using GS method. In addition, the input data of SVR and ELM is consistent with the CNN-LSTM.

**Figure 10:**
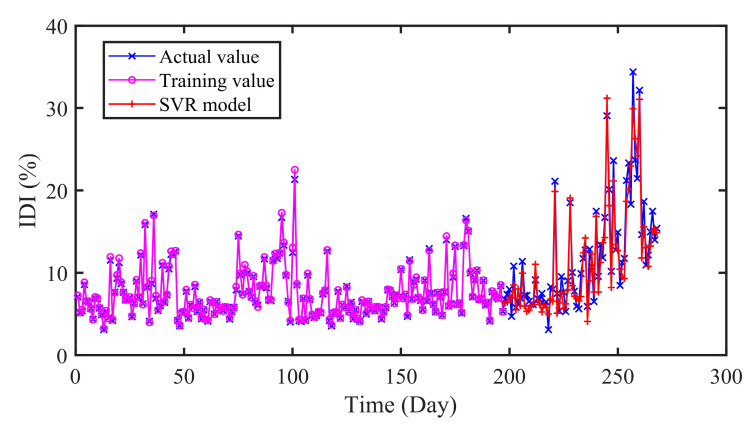
The predicted results of SVR model.

**Figure 11:**
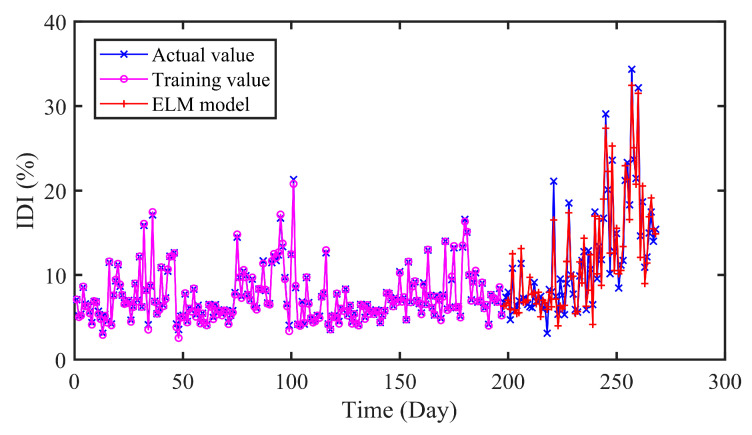
The predicted results of ELM model.

The prediction results of different models are presented in Figure 10 and Figure 11 and Table 5. As can be seen in Table 5, the RMSE and MAE of SVR are 1.9565 and 1.5712 respectively, while that of CNN-LSTM are 1.1588 and 0.8994. Similarly, the results of ELM are 1.9115 and 1.6613. In addition, the R values of SVR and ELM are 0.9214 and 0.9261, which are lower than that of CNN-LSTM 0.9713. On the whole, the proposed CNN-LSTM model achieved minimum values on three indicators, which illustrates the effectiveness of the proposed model.

#### 4.5.2. Comparison of CNN, LSTM and GDAU Prediction

**Figure 12:**
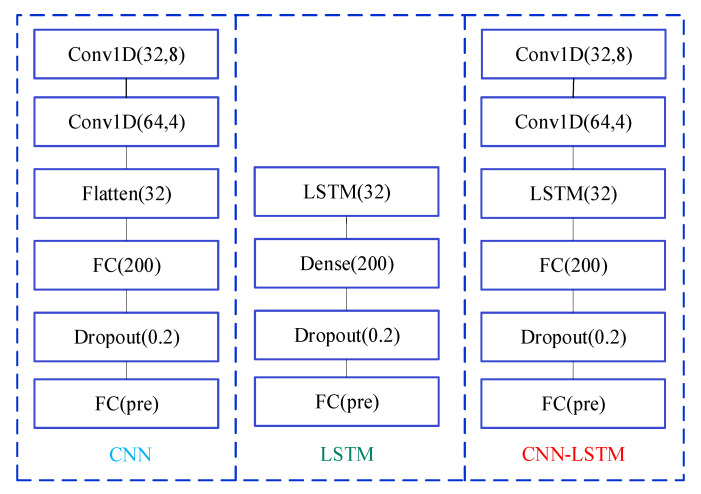
The structure of different models.

Furthermore, we have conducted experiments to compare the proposed model with other deep learning-based models. CNN, LSTM and GDAU are adopted for prediction, and the results are evaluated in four error criteria like RMSE, MAE, MAPE, and R. The comparison models are related strictly according to the comparison principle. The settings of hyper-parameters and experimental conditions are the same as CNN-LSTM. The structure of CNN and LSTM is shown in Figure 12.

**Figure 13:**
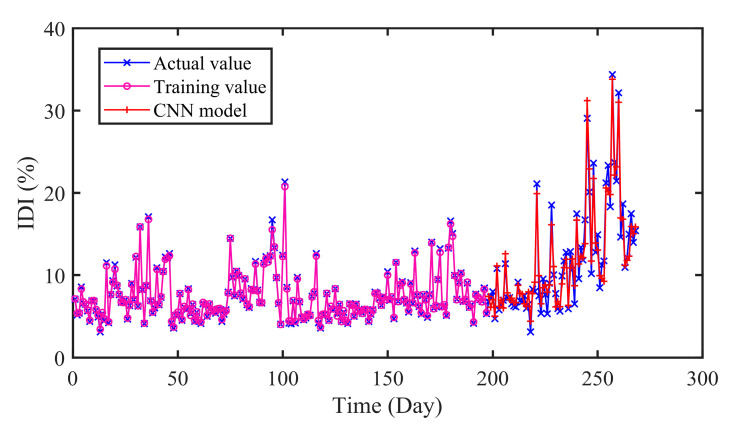
The predicted results of CNN model.

**Figure 14:**
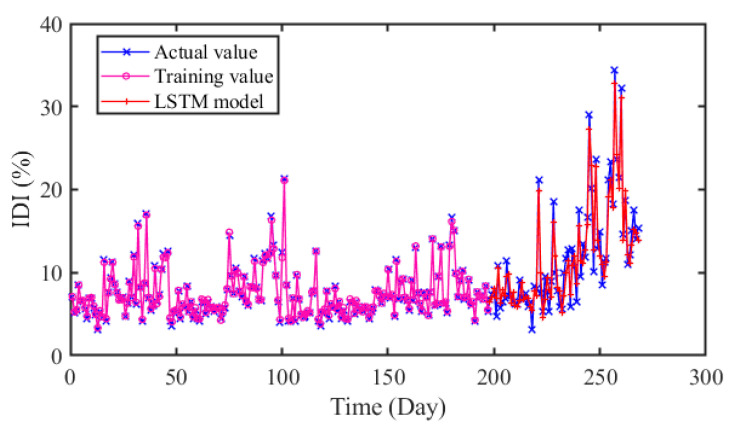
The predicted results of LSTM model.

**Figure 15:**
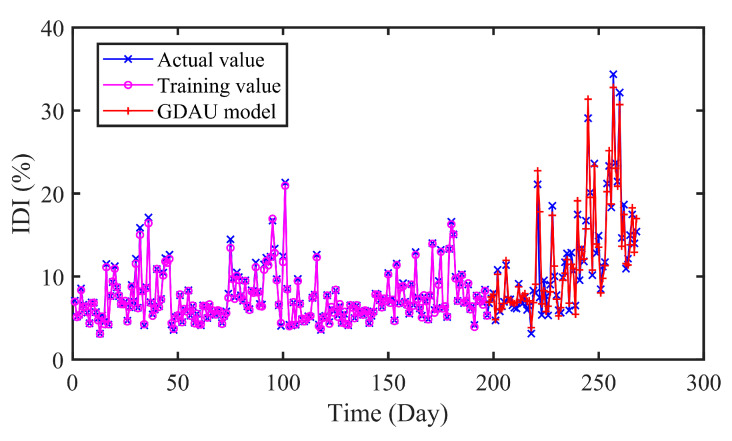
The predicted results of GDAU model.

In Figure 13, Figure 14 and Figure 15, it can be seen that the prediction results of the compared models can approximately fit the actual values with little error. Moreover, the hybrid CNN-LSTM model performs best in RMSE, MAE and MAPE. The RMSE of CNN, LSTM, and GDAU are 1.4233, 1.6166, and 1.1797, respectively. Similarly, the MAE value of CNN-LSTM model is slightly lower than that of compared model. As seen in Table 5, the R value of CNN-LSTM model is 0.9713, which is obviously higher than that of CNN 0.9502 and LSTM 0.9470. The results illustrate the hybrid CNN-LSTM can effectively improve the prediction accuracy by integrating the excellence of CNN in local feature extraction and good expression of LSTM on time series features.

#### 4.5.3. Analysis and Discussion

**Figure 16:**
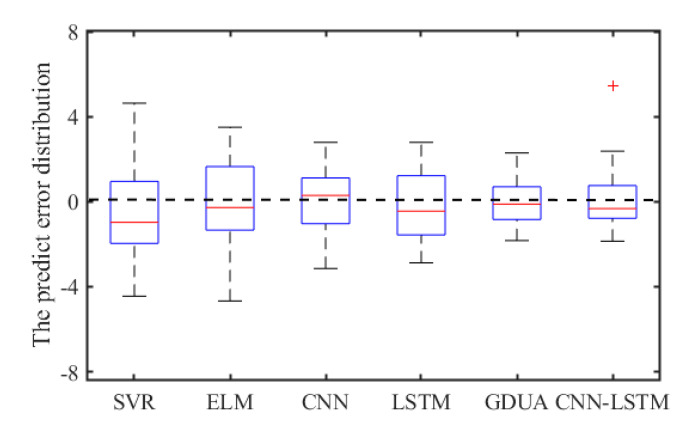
The box plots of error distribution.

**Figure 17:**
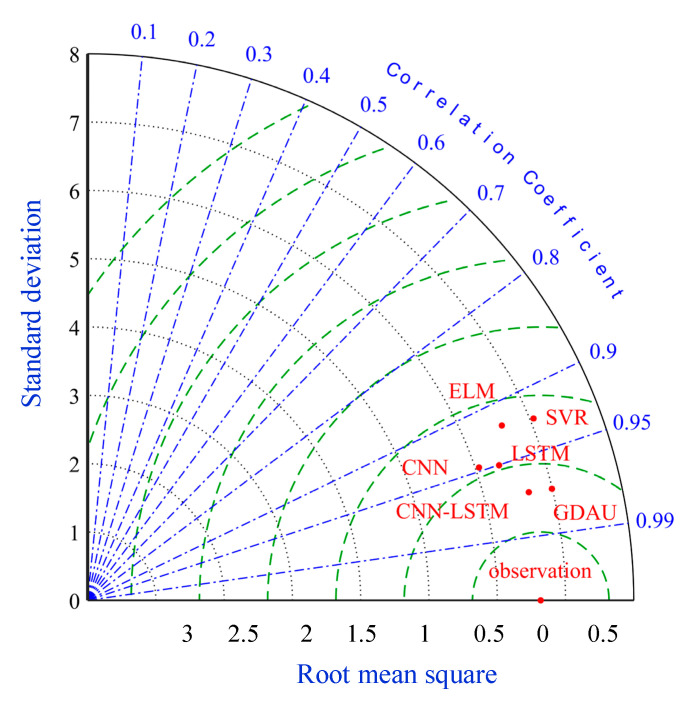
Taylor diagram of all the models.

To compare the performance of various models more intuitively, the results achieved by comparative models are shown in Table 6 and Figure 16 and Figure 17, respectively. The average performance improvement ratio is shown in Table 6 And the error distributions of all models are illustrated in Figure 16. Furthermore, the Taylor diagram is employed as illustrated in Figure 17. The standard deviations, centered root-mean-square and correlations are clearly exhibited.

As can be seen in Table 6, it is clearly indicated that the proposed model performs better than SVR, ELM with the *P*_RMSE_ 40.77% and 39.38%. The same improvement also exists in MAE and MAPE indexes. In addition, the error distributions of SVR and ELM are relatively wide, which means that they cannot achieve uniform precision at almost all points. From Figure 9 and Figure 13, Figure 14 and Figure 15, the prediction results of CNN, LSTM, GDAU and CNN-LSTM models basically consistent with the actual values, expect for the local abrupt change points. Compared with the CNN model and the LSTM model in Table 6, the *P*_RMSE_ of CNN-LSTM model is increased by 18.58% and 28.32%, respectively. Comparing the box plots of errors distribution in Figure 16, the errors of the proposed model are mainly concentrated around 0 and narrow in tiny scales in all the comparative models. It means that the proposed model can achieve uniform precision at almost all points. As shown in Figure 17, the CNN-LSTM model and GDAU model have the roughly same correlation coefficient with observation. However, the proposed model has the same standard deviation as the observation, whereas the GDAU model has more spatial variability considering its larger standard deviation. This means the performance of the GDAU model is also good.

To sum up, the proposed CNN-LSTM model can accurately predict the degradation tendency of PSU. We have confirmed through experiments that the proposed CNN-LSTM is a competitive method for DTP.

## 5. Conclusions

In this paper, a hybrid model is proposed for DTP based on IDI construction and CNN-LSTM. Considering the condition parameters, such as active power (*P*), working head (*H*), and guide vane opening (*G*), the health state model is established with GPR. Then, the IDI that characterizes the degradation level is developed with multi-objects and EW theory. The CNN-LSTM model is applied for DTP with higher accuracy. To illustrate the effectiveness of the proposed model, the vibration dataset and the swing dataset of PSU in China are chosen as case study. The parallel predicting models as SVR, ELM, CNN, LSTM, and GDAU are conducted to highlight the advantages of the proposed model. Comparing the results of all the models, the lowest RMSE, lowest MAE, and lowest MAPE are achieved by the proposed model, which revealed the effectiveness of the CNN-LSTM model for DTP.

However, we paid more attention to the performance of the proposed model and considered the number of parameters comparatively less. In addition, the mixed models have been widely investigated to enhance the predicting performance, where the advantages corresponding to each model could be maximized, thus making full use of each model to deal with different situations. Therefore, the perspectives of our subsequent research work are summarized as follows: (1) some strategies that improve the efficiency of model and reduce the number of parameters will be employed and (2) the combination of multiple predicting models will be the focus of our future work.

## Figures and Tables

**Table 1:** The data monitored from TN8000 system.

The Type of Data	The Type of Sensors	Location
Key phase	Eddy current sensor	Shaft
Swing of upper guide (X, Y direction)	Eddy current sensor	Upper guide
Swing of lower guide (X, Y direction)	Eddy current sensor	Lower guide
Swing of upper bracket (X, Y direction)	Eddy current sensor	Upper bracket
Swing of lower bracket (X, Y direction)	Eddy current sensor	Lower bracket
Vibration of upper guide (X, Y direction)	Low frequency speed sensor	Upper guide
Vibration of lower guide (X, Y direction)	Low frequency speed sensor	Lower guide
Vibration of upper bracket (X, Y direction)	Low frequency speed sensor	Upper bracket
Vibration of lower bracket (X, Y direction)	Low frequency speed sensor	Lower bracket

**Table 2:** The parameters of GPR.

Type	Basis Function	Kernel Function	Kernel Scale	Kernel Sigma	Sigma
Rational Quadratic GPR	Constant	Rational Quadratic	Automatic	Automatic	Automatic

**Table 3:** The entropy and its weight of PDI series.

PDI Series	Lower Guide		Lower Bracket	
	X-Direction	Y-Direction	X-Direction	Y-Direction
Entropy	0.9555	0.9686	0.9857	0.9768
Weight	0.3915	0.2768	0.1260	0.2047

**Table 4:** The specifically structure parameters CNN-LSTM.

Layer	Activation	Kernel Size	Stride	Filter
Input	/	/	/	/
Conv1D	relu	8	1	32
Conv1D	relu	4	1	64
LSTM	relu	/	/	/
FC	relu	/	/	/
FC	linear	/	/	/

**Table 5:** The performance of different models in terms of RMSE, MAE, MAPE and R.

Model	Evaluate Criterion			
	RMSE	MAE	MAPE	R
SVR	1.9565	1.5712	0.1575	0.9214
ELM	1.9115	1.6613	0.1572	0.9261
CNN	1.4233	1.2149	0.1182	0.9502
LSTM	1.6166	1.4022	0.1469	0.9470
GDAU	1.1797	0.9427	0.0836	0.9725
CNN-LSTM	1.1588	0.8994	0.0918	0.9713

**Table 6:** Improved percentages between compared models and the proposed model.

The Proposed vs. Extant Model	*P*_RMSE_ (%)	*P*_MAE_ (%)	*P*_MAPE_ (%)	*P*_R_ (%)
CNN-LSTM vs. SVR	40.77	42.76	41.71	5.42
CNN-LSTM vs. ELM	39.38	45.86	41.60	4.88
CNN-LSTM vs. CNN	18.58	25.97	22.33	2.22
CNN-LSTM vs. LSTM	28.32	35.6	37.51	2.57
CNN-LSTM vs. GDAU	28.32	35.6	37.51	−0.12

## Abbreviations

PSU	Pumped storage unit	RBFNN	Radial basis function neural network
GPR	Gaussian process regression	FC	Fully connected layer
IDI	Integrated degradation index	MSE	Mean square error
CNN	Convolutional neural network	RMSE	Root mean square error
LSTM	Long short term memory	MAE	Mean absolute error
DTP	Degradation tendency prediction	MAPE	Mean absolute percentage error
EW	Entropy weight	SVR	support vector regression
PDI	Performance degradation index	ELM	Extreme learning machine
RNN	Recurrent neural network	GDAU	Gated dual attention unit
LS-SVR	Least squares support vector regression	GS	Grid search
